# Intensive antibiotic treatment of sows with parenteral crystalline ceftiofur and tulathromycin alters the composition of the nasal microbiota of their offspring

**DOI:** 10.1186/s13567-023-01237-y

**Published:** 2023-11-24

**Authors:** Laura Bonillo-Lopez, Pau Obregon-Gutierrez, Eva Huerta, Florencia Correa-Fiz, Marina Sibila, Virginia Aragon

**Affiliations:** 1 Unitat Mixta d’Investigació IRTA-UAB en Sanitat Animal, Centre de Recerca en Sanitat Animal (CReSA). Campus de La Universitat Autònoma de Barcelona (UAB), 08193 BellaterraBarcelona, Catalonia Spain; 2 IRTA, Programa de Sanitat Animal. Centre de Recerca en Sanitat Animal (CReSA), Campus de la Universitat Autònoma de Barcelona (UAB), Bellaterra, 08193 Barcelona, Catalonia Spain; 3 WOAH Collaborating Centre for the Research and Control of Emerging and Re-Emerging Swine Diseases in Europe (IRTA-CReSA), Bellaterra, 08193 Barcelona, Spain

**Keywords:** Nasal microbiota, antibiotics, sows, piglets, bacterial transmission

## Abstract

The nasal microbiota plays an important role in animal health and the use of antibiotics is a major factor that influences its composition. Here, we studied the consequences of an intensive antibiotic treatment, applied to sows and/or their offspring, on the piglets’ nasal microbiota. Four pregnant sows were treated with crystalline ceftiofur and tulathromycin (CT_sows_) while two other sows received only crystalline ceftiofur (C_sows_). Sow treatments were performed at D-4 (four days pre-farrowing), D3, D10 and D17 for ceftiofur and D-3, D4 and D11 for tulathromycin. Half of the piglets born to CT_sows_ were treated at D1 with ceftiofur. Nasal swabs were taken from piglets at 22–24 days of age and bacterial load and nasal microbiota composition were defined by 16 s rRNA gene qPCR and amplicon sequencing. Antibiotic treatment of sows reduced their nasal bacterial load, as well as in their offspring, indicating a reduced bacterial transmission from the dams. In addition, nasal microbiota composition of the piglets exhibited signs of dysbiosis, showing unusual taxa. The addition of tulathromycin to the ceftiofur treatment seemed to enhance the deleterious effect on the microbiota diversity by diminishing some bacteria commonly found in the piglets’ nasal cavity, such as *Glaesserella*, *Streptococcus*, *Prevotella*, *Staphylococcus* and several members of the *Ruminococcaceae* and *Lachnospiraceae* families. On the other hand, the additional treatment of piglets with ceftiofur resulted in no further effect beyond the treatment of the sows. Altogether, these results suggest that intensive antibiotic treatments of sows, especially the double antibiotic treatment, disrupt the nasal microbiota of their offspring and highlight the importance of sow-to-piglet microbiota transmission.

**Supplementary Information:**

The online version contains supplementary material available at 10.1186/s13567-023-01237-y.

## Introduction

The animal microbiota is defined as the ecological community of microorganisms found in different body sites, which are considered their niches [1]. Several studies have reported positive effects and functions that the microbiota provides to their hosts, including metabolic benefits, immune system maturation, protection against pathogens and other physiological functions [2, 3]. Due to the importance of the microbiota functions, the stability of the bacterial community is crucial for the health and welfare of their hosts [3–6]. In general, the gut has been the main niche targeted in microbiota studies, but less-studied microbiomes have also proven to be highly important in animal health, as for example, the nasal microbiota [7, 8]. This microbiota is the first protection against colonization by respiratory pathogens, which need to overcome this barrier to systemically infect the host [9]. In fact, it has been demonstrated that the nasal microbiota plays a role in the development of several swine respiratory diseases [8, 10–13].

One of the first sources of microbiota for the piglets are their mothers, firstly through exposure to the vaginal tract during birth and, later, by colostrum and milk intake together with the exposure to their faecal and skin microbiomes [14, 15]. Hence, the transmission of microorganisms from the dam is determinant for the early microbial acquisition by the piglet and is crucial for the proper development of their microbiome and immune system [3, 14, 16]. Among early colonizers, *Glaesserella parasuis*, *Streptococcus suis* and *Mycoplasma hyorhinis*, are pathobionts found to be transmitted from sows to their offspring [17–19].

Weaning, normally done in commercial farms at 3–4 weeks of age [20], is a stressful moment in piglets’ lives that has a big impact on their microbiota diversity and composition affecting also their health status [10]. Changes caused by the separation from the sows [14], change to solid feed [21], different environmental conditions [14], or vaccination programs [22] have been shown to contribute to increasing the risk of disease development, and to impact the nasal microbiota of piglets at this stage. Therefore, knowledge on the factors involved in the establishment and those that alter the swine microbiota is key in pig health. Among these factors, the use of antibiotics is one of the most concerning ones, not only for their association with antimicrobial resistances, but also for their deleterious effects on the microbiota [2, 3, 10]. In farms, sows are sometimes treated with antibiotics to control pathogen transmission to their offspring [23]; however, these treatments may have an impact on the natural early colonization of their offspring. Indeed, there is a need to reduce the use of these substances in animal production [24].

Ceftiofur and tulathromycin, are two antibiotics used in animal production against respiratory diseases in swine, cattle, and other animals [25–31]. Ceftiofur is a broad-spectrum antimicrobial that inactivates penicillin-binding proteins (PBPs) and interferes with the cross-linkage of peptidoglycan chains necessary for building the bacterial cell wall, resulting in the weakening of this structure and the consequent lysis of the bacterial cells [32]. Tulathromycin is a macrolide that inhibits bacterial protein synthesis by binding to the ribosomal 50S subunit, which results in a bacteriostatic and bactericidal activity. Due to its positive charge, this drug has a preferential activity against Gram-negative bacteria and *Mycoplasma spp*. [26, 33]. It has been shown that the administration of crystalline ceftiofur or tulathromycin, among others antibiotics, in 8-week-old piglets has an impact in the nasal microbiota, changing the microbial populations at both phylum and genus level [34]. Despite the effect of the use of β-lactams on the nasal microbiota has been assessed in piglets and sows [34–36], to our knowledge, the effect of the co-administration of crystalline ceftiofur and tulathromycin on the bacterial transmission from sows to piglets has not been studied.

The goal of this study was to compare the effect of two intensive antibiotic treatments given to sows (crystalline ceftiofur alone or together with tulathromycin) on the nasal microbiota of their piglets. Moreover, we also aimed to assess if the effect of the double antibiotic treatment in sows was enhanced by an additional treatment of crystalline ceftiofur on piglets.

## Materials and methods

### Experimental design and sampling

Animal experimentation was performed following proper veterinary practices, in accordance with European (Directive 2010/63/EU) and Spanish (Real Decreto 53/2013) regulation and with the approval of the Ethics Commission in Animal Experimentation of the Generalitat de Catalunya (Protocol Number 11150).

Six pregnant sows were moved to IRTA-CReSA facilities 2 weeks pre-farrowing. Two sows were treated with 15 mL of 5 mg/kg crystalline ceftiofur (C_sow_) four days before farrowing (D-4), and at D3, D10 and D17 and four sows received the same treatment in addition to 6 mL of 2.5 mg/kg tulathromycin (CT_sows_) at D-3, D4 and D11 (Table 1).

**Table 1:** **Study design**: groups, number of animals and treatments administrated to sows and piglets: (crystalline ceftiofur + tulathromycin treated sows, non-treated piglets, CT_sow_N_piglet_; crystalline ceftiofur + tulathromycin treated sows, crystalline ceftiofur treated piglets, CT_sow_C_piglet_; crystalline ceftiofur treated sows, non-treated piglets, C_sow_N_piglet_)

Group	Number of sows	Sow treatment day		Number of piglets	Piglet treatment day
		Crystalline ceftiofur	Tulathromycin		Crystalline ceftiofur
CT_sow_N_piglet_	*N* = 2	D-4, D3, D10 and D17	D-3, D4 and D11	*N* = 7	–
CT_sow_C_piglet_	*N* = 2	D-4, D3, D10 and D17	D-3, D4 and D11	*N* = 8	D1
C_sow_N_piglet_	*N* = 2	D-4, D3, D10 and D17	-	*N* = 11	–

Farrowing was induced by injecting 1 mL of 0.075 mg/mL Veteglan to the sows. At birth (D0), piglets took colostrum from their biological mothers for at least 2 h and were cross-fostered to avoid the bias from the sow to their litter. Afterwards, piglets born to sows from CT_sows_ were randomly distributed in two groups: group CT_sow_C_piglet_ (*n* = 8), where animals were treated at D1 with a dose of 0.1 mL 5 mg/kg of crystalline ceftiofur, and group CT_sow_N_piglet_ (*n* = 7), in which piglets remained untreated (Table 1). Piglets born to sows from C_sow_ did not receive any antibiotic treatment (C_sow_N_piglet,_ *n* = 11). Piglets from all groups were observed until weaning for clinical signs.

Nasal sampling was performed with thin aluminium cotton swabs on both nostrils before (D-7) and after (D0) the first antibiotic administration on sows and on piglets at weaning (D22-D24). Moreover, nasal swabs from six age-matched animals (21 days of age) from healthy farms were sampled as a control for reference value of the total bacterial load. All nasal swab samples from piglets and sows were resuspended in 500 µL of PBS and stored at −80 °C until processed.

### DNA extraction and PCR/qPCR testing

DNA extraction from all nasal swabs taken from sows and piglets was performed using the NucleoSpin Blood kit (Machinery Nagel, GmbH & Co, Düren; Germany) following the manufacturer’s protocol instructions. DNA concentration was measured using absorbance at 260 nm (A_260_) with BioDrop DUO (BioDrop Ltdre). Moreover, to assess the total bacterial load present in the samples, a real-time (RT) qPCR targeting the 16S rRNA gene was performed. This reaction was prepared in a volume of 20 μL consisting in 2 μL of the template DNA and 18 μL of Femto Bacterial qPCR Premix (Femto Bacterial DNA Quantification Kit, Zymo Research) and run following the manufacturer’s protocol. Samples were quantified using different dilutions of DNA from *Escherichia coli* strain JM109 provided as a standard in the kit. Following manufacturer’s recommendations, samples were considered negative with a cycle threshold (Ct) > 33. Graphpad 8.3 (538) Prism software (Dotmatics, San Diego, CA, USA) was used for statistical analysis. Wilcoxon matched-pairs signed rank test [37] was used to compare the bacterial quantity in sow samples before and after the antibiotic treatment. Kruskal–Wallis multiple comparison [38] with Benjamini, Krieger and Yekutieli post-hoc test [39] was used to compare the bacterial quantity among the three different groups of piglets. *P* < 0.05 were considered statistically significant.

The presence of early colonizers (*G. parasuis, S. suis* and *M. hyorhinis*) in the piglet’s nasal cavities was tested by specific PCR/qPCR to confirm the possible reduction in bacterial transfer. The PCR used for the detection of *G. parasuis* allows the discrimination between virulent and non-virulent strains [40]. For *S. suis*, a conventional PCR was performed following a previously described protocol [41] modifying the annealing temperature (from 55 °C to 60 °C) and using 1 U GoTaq polymerase (Promega). Amplicons from both conventional PCRs were analysed by electrophoresis on 2% agarose gels. *M. hyorhinis* qPCR was performed using a previously described protocol [42] modifying the number of cycles (from 35 to 40). Samples were considered negative when the Ct > 39 cycles.

### 16S rRNA gene sequencing and microbiota analysis

From the total extracted DNA from piglets’ nasal swabs at D22-D24, the 16S rRNA gene libraries were prepared and sequenced in two runs with Illumina MiSeq pair-ended (2X300 bp, MS-102–2003 MiSeq Re-agent Kit v2, 500 cycle) at the Servei de Genòmica, Universitat Autònoma de Barcelona (Spain). The amplicon sequences corresponding to V3-V4 hypervariable regions of the 16S rRNA gene were demultiplexed and used as input for downstream bioinformatics analyses.

The analyses of the nasal microbiota of piglets were performed using quantitative insights into microbial ecology (QIIME) 2 software version 2022.2 [43]. First, raw reads were imported in QIIME2 and quality assessed using *q2 demux * plugin. Primers were trimmed out from forward and reverse reads using Illumina V3V4 adapter sequences with *q2 cutadapt* plugin. DADA2 [44] was used to denoise the reads, i.e. quality-filtering, read-merging and chimera removal, and sort them into Amplicon Sequence Variants (ASVs) for each run. Additionally, low-quality 3’ end positions were truncated from the reads. After, ASVs not matching the 88% pre-clustered Greengenes database Vs. 13.8. [45, 46] at 65% identity and 50% query coverage were filtered out using VSEARCH [47] within *q2 quality control* plugin [48], to eliminate spurious nonprokaryotic features (unspecific contaminants). Furthermore, non-bacterial sequences classified as *Archaea*, *Chloroplast* or *Mitochondria* were also removed from the data set. Since the reads included in this analysis were obtained in two different runs, after all these denoising and filtering steps, all data was merged for the downstream analysis. Curated merged sequences were aligned with MAFFT [49] and hypervariable positions were masked [50] with *q2 alignment* plugin. Finally, the phylogenetic tree was built using Fastree [51]. For the first diversity analysis, the depth used was 17,348, corresponding to the lowest sample depth evaluated through rarefaction curves [52].

The farm core-microbiota was calculated at genus level considering nasal samples of same-aged healthy animals from Spanish (*n* = 39) and British (*n* = 18) farms from previous studies [5, 8]. All genera present in at least 80% of all farm samples were considered as farm core microbiota and hence, common members of the swine nasal microbiota. In this study, we excluded all ASVs whose classification did not match the defined genera using the QIIME2 software options to filter the data. As the core-microbiota also contained taxa with unresolved classification to genus level (I, e, *Bacteroidales* or *Moraxellaceae*), we also kept those ASVs with such unresolved classifications. After eliminating the ASVs absent from the farm core-microbiota, the lowest sample depth to be used in the diversity analyses was 3,629.

Alpha diversity (diversity found within each sample) was estimated with Chao [53] and Shannon [54] indexes, and the significance between groups was computed by pairwise non-parametric t-tests (999 random permutations) with *q2 diversity alpha-group-significance* plugin [38]. The distance matrices to estimate beta diversity (diversity between samples) were computed using *q2 core-metrics plugin* and used to perform principal coordinate (PCoA) analysis [55, 56]. Jaccard [57] and Bray Curtis [58] dissimilarity measures were used to estimate beta diversity qualitatively and quantitatively respectively and visualized using Emperor [59]. To quantify the group variation of the variables under study (R^2^), we used Adonis function from Vegan package in R software [60], where the significance was calculated by PERMANOVA pairwise test (999 random permutations) using *q2 diversity beta-group-significance* plugin [61]. PERMANOVA test was also performed to estimate the significance of the clustering on both qualitative and quantitatively distance matrices.

ASVs were taxonomically classified with scikit-learn (Python module for machine learning) using a naïve Bayes classifier [62], previously trained against V3-V4 regions from 16S rRNA gene with Greengenes database vs. 13.8 pre-clustered at 99% sequence identity, to improve its performance as suggested by Werner et al. [63]. To perform differential abundances estimation, we used two complementary approaches to compare the groups: discrete False-Discovery Rate (dsf-dr) [64] and Analysis of Compositions of Microbiomes with Bias Correction (ANCOM-BC) [65]. In all tests, *P* values lower than 0.05 were considered significant.

## Results

### Antibiotic treatment of sows reduces the microbial transfer to their offspring

**Figure 1:**
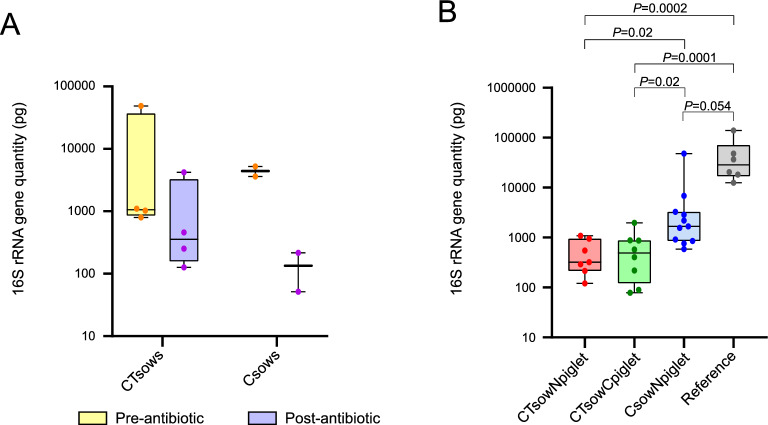
**Quantitative PCR of 16S rRNA gene in nasal swabs. A** 16S rRNA gene quantity (pg) detected by qPCR in nasal swabs taken from sows before (Pre-antibiotic, in yellow) and after (Post-antibiotic, in purple) first administration of their respective antibiotic treatments: crystalline ceftiofur + tulathromycin sows (CT_sows_) and crystalline ceftiofur sows (C_sows_). Each dot corresponds to one animal. **B** 16S rRNA gene quantity (pg) detected by qPCR in nasal swabs from piglets of the groups under study: non-treated piglets born to ceftiofur + tulathromycin treated sows (CT_sow_N_piglet_, red); ceftiofur treated piglets born to ceftiofur + tulathromycin treated sows (CT_sow_C_piglet_, green); non-treated piglets born to ceftiofur treated sows (C_sow_N_piglet_, blue); and the reference group of age-matched farm piglets (grey). Each dot corresponds to one animal. Significant *P* values are shown in upper bars.

To investigate whether the antibiotic treatments could reduce the bacterial transmission from sow to piglets, DNA was extracted and quantified from nasal swabs from all sows and piglets of the study. In sows, we found that the total amount of DNA estimated using the absorbance at 260 nm (A_260_) was numerically lower (mean ± standard deviation, SD) after the first antibiotic treatment in both treated groups (CT_sows_ 202 ± 16.1 ng and C_sows_ 203 ± 6.8 ng) than before this treatment was applied at D-7 (CT_sows_ 500 ± 362.6 ng and C_sows_ 321 ± 132.3 ng). Indeed, the bacterial load (quantity of 16S rRNA gene) was also numerically reduced after the first antibiotic treatment in both CT_sows_ and C_sows_ groups (Figure  1A). However, these differences were not statistically significant for the CT_sows_ group (Wilcoxon matched-pairs signed rank test, *P* = 0.6250) and not possible to confirm for the C_sows_ group due to the low group size (*n* = 2). Nevertheless, when considering all treated sows together, the bacterial load after antibiotic treatment was significantly reduced (Wilcoxon matched-pairs signed rank test, *P* = 0.0260).

In piglets, the amount of total DNA extracted from nasal swabs (D22-24) measured at A_260_ was lower in samples taken from CT_sow_N_piglet_ (433.8 ± 221.4 ng), CT_sow_C_piglet_, (432 ± 229 ng) and C_sow_N_piglet_ (1155 ± 802 ng) than from six age-matched healthy farm animals used as a reference control (1443.4 ± 1250.8 ng). Again, this result is in agreement with the total bacterial load (16S rRNA gene quantification) that also showed a reduction due to the antibiotic treatment (Figure 1B). All the piglets born to treated sows showed a reduced bacterial load compared with a group of the six age-matched healthy farm animals. The treatment of the sows with the combination of ceftiofur and tulathromycin caused a more pronounced reduction in bacterial load in their offspring than the treatment with only ceftiofur (Kruskal–Wallis test Multiple comparisons adjusting *P* values with a Benjamini, Krieger and Yekutieli correction method). On the other hand, the extra treatment performed to the piglets with ceftiofur did not result in higher decrease in bacterial load in their nasal cavities (*P* = 0.9414, CT_sow_N_piglet_ vs CT_sow_C_piglet_; Figure 1B).

The presence of typical pathobionts from the swine nasal microbiota was analysed to evaluate the effect of the antibiotic treatment on the transfer dynamics from sows to piglets. All nasal swabs from sows taken before and after the antibiotic treatment were negative to *S. suis* and *G. parasuis* PCRs*,* as well as *M. hyorhinis* qPCR. Similarly, all nasal swabs from piglets were negative for *S. suis* and for *M. hyorhinis* by PCR/qPCR. On the other hand, CT_sow_N_piglet_ piglets were negative for *G. parasuis*, but 4 out of 11 (36%) piglets of C_sow_N_piglet_ were positive for non-virulent *G. parasuis* strains and 1 out of 8 (12.5%) piglets of CT_sow_C_piglet_ was positive for virulent *G. parasuis* strains.

### The antibiotic treatment on sows altered the nasal microbiota composition of the piglets

In order to assess how the antibiotic treatment impacted the composition of the nasal microbiota of the piglets, we performed 16S rRNA gene sequencing analysis. After raw read pre-processing, a final number of 6666 different ASVs were obtained (total frequency of 1374806), with a mean frequency per sample of 52877.15. All ASVs were classified at different taxonomic levels to characterize the nasal microbiota composition of the piglets. Surprisingly, a large percentage of the microbial community was represented by the orders *Burkholderiales* and *Rhizobiales*, with a mean abundance ± SD across all groups of 33.7% ± 17.4 and 11.4% ± 9.3, respectively. The most relatively abundant genera within these orders were *Ralstonia*, *Afipia* and *Hyphomicrobium*, indicating an overabundance of environment-associated taxa. Other relatively abundant taxa belonged mainly to the orders *Clostridiales*, *Pseudomonadales*, *Bacteroidales*, *Lactobacillales* and *Pasteurellales*, including typical nasal-associated genera such as *Prevotella*, *Streptococcus*, *Acinetobacter*, *Ruminococcaceae (uncl.)*, *Lachnospiraceae (uncl.)* and *Glaesserella* (see Additional file 1 for the whole composition at genus level and Additional file 2 for the most relatively abundant taxa at order level).

**Figure 2:**
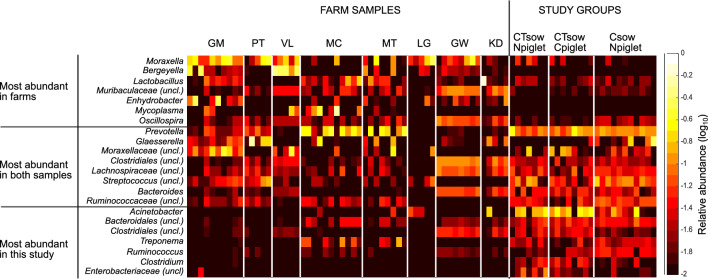
**Comparison of the most abundant taxa in the study groups and healthy farms.** Relative abundances (log scaled) of the top 15 most prevalent genera found in nasal cavities of piglets from the different groups of this study and in age-matched animals from farms from the studies of Correa-Fiz et al. [5, 8]. Genera have been labelled as found between the most abundant in farms, this study groups, or both. Farms are labelled withs their original ID from their respective studies. Abundances in samples from this study are shown per group: non-treated piglets born to ceftiofur + tulathromycin treated sows (CT_sow_N_piglet_); ceftiofur treated piglets born to ceftiofur + tulathromycin treated sows (CT_sow_C_piglet_); non-treated piglets born to ceftiofur treated sows (C_sow_N_piglet_).

Since taxa not commonly found in the nasal microbiota was detected in relatively high abundance (probably due to the low bacterial load in the nasal cavity of these animals caused by the antibiotic treatments), we filtered out the ASVs classified as these uncommon taxa and continued the analyses with only those ASVs belonging to the core-microbiota of healthy farm piglets (see methods). The nasal core-microbiota from farm piglets represented a mean of 30.57% (± 30.46%), 21.89% (± 20.26%), and 39.21% (± 17.13%) of the total abundance for CT_sow_N_piglet_, CT_sow_C_piglet_ and C_sow_N_piglet_ groups, respectively. The final filtered data consisted of 2319 ASVs (total frequency of 385391), with a mean frequency per sample of 14822.7. After filtering, the nasal microbiota was dominated by genera within the orders *Clostridiales* (general abundance of 35.2 ± 5.8%), mainly composed by the families *Lachnospiraceae* and *Ruminococcaceae*; *Bacteroidales* (22.2 ± 6%), with *Prevotella* and *Bacteroides* as the most prevalent genus; *Pseudomonadales* (16 ± 9.6%) with genera such as *Acinetobacter* and an unclassified member from the *Moraxellaceae* family*; Lactobacillales* (8.65 ± 5%), with *Streptococcus* and *Lactobacillus* within the most relatively abundant genera; *Enterobacteriales* (4.45 ± 4.5%), with *Escherichia* as the most abundant genus; and *Pasteurellales* (3 ± 7%), mainly represented by *Glaesserella*. The abundances of the genera after filtering are detailed in Additional file 3, and the most relatively abundant genera are represented in Additional file 4. With the aim to quantitatively compare the microbiota composition from animals from this study with those from farms used as reference core-microbiota, we focused on the most abundant taxa in each type of samples (Figure 2). Eight of the most abundant genera were shared between farms and the groups under study. On the contrary, some typical swine nasal colonizers detected among the most abundant genera in farm samples, such as *Moraxella*, *Bergeyella* or *Lactobacillus*, were not found among the most abundant taxa in this study. At last, we detected some genera that were highly represented in the samples of this study while found in low abundance in farms, including *Acinetobacter*, *Clostridium* or *Treponema*.

### The sow antibiotic treatments differentially altered the nasal microbiota diversity of the piglets

**Figure 3:**
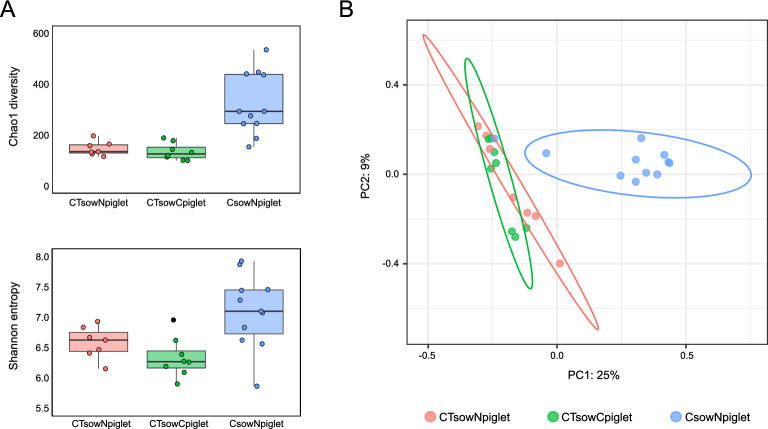
**Alpha and beta diversity analysis of the groups under study.** Non-treated piglets born to ceftiofur + tulathromycin treated sows (CT_sow_N_piglet_, red); ceftiofur treated piglets born to ceftiofur + tulathromycin treated sows (CT_sow_C_piglet_, green); non-treated piglets born to ceftiofur treated sows (C_sow_N_piglet_, blue). **A** Alpha diversity boxplots estimated with Chao1 and Shannon indexes. Each dot represents a sample. Dots corresponding to outlier simples are coloured in black. **B** Beta diversity PCoA analysis computed with Bray–Curtis dissimilarity index, of the groups under study. Each dot represents a sample. Ellipses of confidence are calculated using Euclidean distances within the samples of each group.

A diversity analysis was performed to understand whether the antibiotic treatments had a different impact on the nasal microbiota of piglets. Higher richness and evenness (Chao1 and Shannon) were found in the C_sow_N_piglet_ group compared to the groups of piglets born to sows treated with the two antibiotics (*P* < 0.05, Figure 3A). In the beta diversity analysis, the C_sow_N_piglet_ group clustered as a different community when it was compared to the two CT_sow_ groups (Jaccard and Bray–Curtis, Figure 3B, PERMANOVA *P* = 0.001). On the contrary, differences between CT_sow_N_piglet_ and CT_sow_C_piglet_ groups were not significative in both qualitative and quantitative analyses.

**Figure 4:**
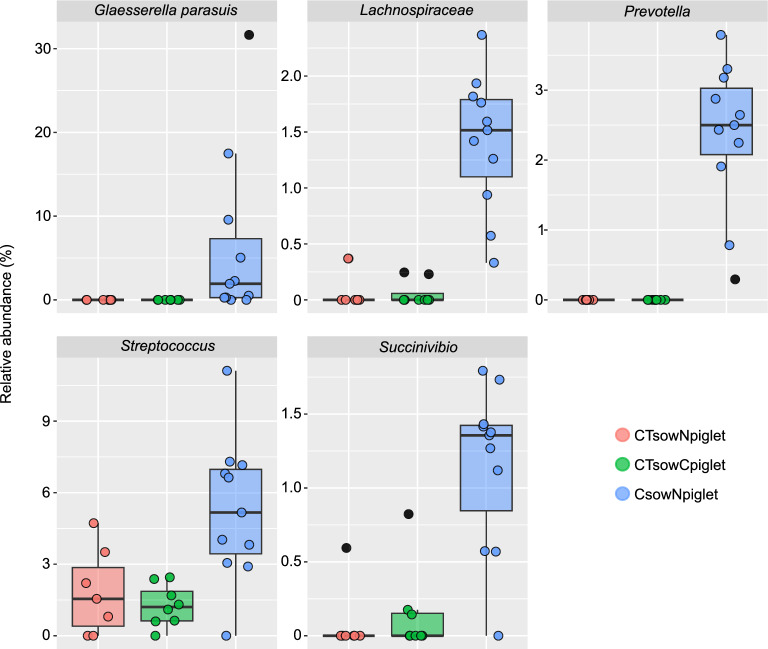
**Differently abundant ASVs between CT**_**sow**_** and C**_**sow**_** piglets.** Top 5 most relatively abundant ASVs within all the differentials found with ANCOM-BC and DSFDR when comparing non-treated piglets born to ceftiofur + tulathromycin treated sows (CT_sow_N_piglet_, red) and non-treated piglets born to ceftiofur treated sows (C_sow_N_piglet_, blue). The abundances of these ASVs in ceftiofur treated piglets born to ceftiofur + tulathromycin treated sows (CT_sow_C_piglet_) are shown too (green). Dots corresponding to outlier samples are coloured in black. All the differently abundant ASVs are listed in Additional file 6.

To study the effect of the antibiotic treatments when applied only to sows, CT_sow_N_piglet_ and C_sow_N_piglet_ groups were compared, eliminating the treatment of piglets as a potential confounding factor. The beta diversity was significantly different between these groups, where the effect size was estimated to be 13.7% and 20.8% for qualitative and quantitative (Additional file 5A) analyses, respectively (Adonis R^2^ value, *P* = 0.001). Accordingly, several differently abundant ASVs were found between these two groups (ANCOM-BC and dsf-dr, Additional file 6). C_sow_N_piglet_ group showed increased abundances of different ASVs belonging to *Bacteroides* and *Prevotella* (*Bacteroidales*); *Staphylococcus* (*Bacillales*); *Streptococcus* (*Lactobacillales*); *Lachnospiraceae* and *Ruminococcaceae* (*Clostridiales*); *Glaesserella* (*Pasteurellales*); *Acinetobacter* (*Pseudomonadales*); and *Succinivibrio* (*Aeromonadales*), among others. The top five most relatively abundant differential ASVs are shown in Figure 4. Despite this finding at ASV level, similar differences were not reflected at higher taxonomic levels, as very few differences between families, genera were found (see Additional file 7).

The effect of the additional treatment with ceftiofur of the piglets born to sows treated with the two antibiotics was evaluated by comparing the microbiota composition of piglets born to CT_sows_ (CT_sow_N_piglet_ and CT_sow_C_piglet_). The clustering of the samples according to the treatment of piglets was not significant in either the qualitative or quantitative beta diversity analysis (PERMANOVA *P* > 0.05, Additional file 5B), confirming that the treatment of the sows was the major driver of the changes observed in the piglets (Figure 3). Accordingly, no differentially abundant taxa were found between the two groups at any taxonomic level, except for one single low abundant ASV classified as *Streptococcus* (0.8% in CT_sow_N_piglet_ and 0.01% in CT_sow_C_piglet_ groups).

## Discussion

Our study shows that intensive antibiotic treatment in sows severely affected the microbial communities in the piglets’ nasal cavities. This effect was more pronounced when using a combination of crystalline ceftiofur and tulathromycin than only using crystalline ceftiofur. Sow treatments affected the bacterial transfer from sows to piglets, which showed a nasal microbiota with reduced alpha diversity and decreased populations of commonly found swine nasal colonizers. The addition of an extra administration of ceftiofur to newborn piglets had no further effect.

The low transfer of microbiota from the sows seemed to increase the detection in the piglets’ nasal cavities of uncommon bacteria for this niche, with taxa from the orders *Burkholderiales* and *Rhizobiales* (*Ralstonia*, *Afipia* and *Hyphomicrobium*) among the most abundant ones. These microorganisms are not found in the nasal microbiota of pigs under standard farm conditions and are unlikely to be part of the swine nasal microbiota. These taxa are often associated with plants, as symbionts or pathogens [66, 67] and they probably came from the food or the extraction kit in the case of *Ralstonia*, as it has been shown in other studies [68]. The detection of environmental microbes in high relative abundance could be caused by the reduced presence of professional colonizers, creating a low-biomass environment prone to be colonized by transit microorganisms, as it has been previously observed [69]. In agreement, during the pre-processing of raw reads, we found chloroplast and mitochondrial 16S sequences in unusual high abundances (9.5% and 4.3%, respectively) in comparison with the farm animals evaluated in this study, as well as in previous studies (0.07% and 0.03%, respectively) [5, 8].

Besides the unusual microbes described above, the rest of the microbiota was constituted of taxa previously found in the swine respiratory microbiota [5, 7, 8, 31, 70–72], which includes aerobic taxa as well as gut-associated anaerobic taxa that are commonly found and have been shown to be active in the pig’s nose [73]. Nevertheless, all of them were initially detected in a very low abundance (considering also the 16S rRNA gene qPCR) and were represented by an unusual low quantity of ASVs. Altogether, these results suggest that the antibiotic treatment had a drastic effect on the usual nasal colonizers. This is in agreement with several studies assessing the effect of antibiotic treatments on the microbiota [5, 31, 34, 36, 74]. As Mou et al. have reported, pig nasal microbiota shifted in response to the broad-spectrum antibiotic oxytetracycline treatment, normally used to treat respiratory bacterial diseases in swine (including *Mycoplasma*, *Pasteurella* and *Glaesserella*). They determined that oxytetracycline administered orally had a major impact in the diversity and disturbance of the microbiota than the intramuscular route [31]. In the present study, we only assessed the intramuscular administration and observed that ceftiofur and tulathromycin administered by this route was enough to severely disturb the nasal microbiota and avoid the bacterial transfer from sow to piglet. In particular, sow antibiotic treatment reduced drastically the bacterial transfer of natural nasal microbiota members, including the three pathobionts *G. parasuis*, *M. hyorhinis* and *S. suis*. Although *G. parasuis* was not found in any of the sows, it is known that the level of this bacterium in nasal swabs from sows is sometimes too low to be detectable [17]. The results obtained by PCR in piglets could be explained if the animals carried *G. parasuis* strains sensitive to tulathromycin but resistant to ceftiofur. This could be attributed to the presence of plasmids that bear resistances to β-lactams, as the ROB-1 β-lactamase reported in the pB1000 and the pJMA-1 plasmids. These plasmids were found in strains recovered from the nasal cavities from healthy animals and considered non-virulent strains [75]. In the case of *S. suis*, the transmission of this pathogen from sow to piglet seems to be prevented. However, we cannot discard the presence of *S. suis* in tonsils, since we have not analysed this niche, which is preferentially colonized by this bacterium [71]. Similarly, *M. hyorhinis* colonization in piglets seems to have been prevented, but it is also possible that this colonization could occur later in life [18].

In farms, the antibiotic treatments given to the sows are intended to reduce pathogen transmission to the piglets. However, our results indicate that these interventions can also have negative consequences, since the dysbiosis produced by these drugs could facilitate pathogen colonization, with the consequent higher risk of infection. In fact, we detected in piglets from treated sows some potentials pathogens such as *Acinetobacter* [76, 77], *Clostridium* [78] or *Treponema* [79] that were not found in the farm samples. In good health status farms, the colonization of these pathogens is probably controlled by the exclusion provided by the normal nasal microbiota. Other explanation could be the selection of resistant strains from these potential pathogens. In agreement, *Acinetobacter* was detected together with eighteen different antibiotics in the groundwater of areas affected by swine farming [76]. Moreover, the poorly establishment of the microbiota in the early ages of the animal live could determine the proper maturation of their immune system [80].

Several studies have demonstrated that the use of antimicrobial drugs in sows have an important impact on the establishment of the microbiota in the firsts weeks of life of their offspring [7], and that this effect lasted longer when administered to the sows than directly to their piglets [36]. In the present study, the long-term effect was not evaluated, as we only had piglet nasal swabs from one timepoint (D22-24). It would have been very interesting to elucidate the impact of the transitory effect of these antimicrobials in an extended period of time.

In conclusion, our results evidence the importance of the maternal microbiota in the establishment of the respiratory microbiota of piglets, which can have a subsequent impact in the control of potential pathogens. This should be taken into consideration when setting treatment plans and routines in swine industry.

### Supplementary Information

**Additional file 1 Relative abundance of the genera found in the nasal microbiota of the piglets included in this study.** Non-treated piglets born to ceftiofur + tulathromycin treated sows (CT_sow_N_piglet_); ceftiofur treated piglets born to ceftiofur + tulathromycin treated sows (CT_sow_C_piglet_); non-treated piglets born to ceftiofur treated sows (C_sow_N_piglet_). Genera with global relative abundance below 0.5% are summed as low abundant.

**Additional file 2 Relative abundance (%) of the top-10 most abundant orders in the piglet’s nasal microbiota.** Microbiota composition is shown for each group included in the present study at order level. CT_sow_N_piglet_, non-treated piglets born to ceftiofur + tulathromycin treated sows; CT_sow_C_piglet_, ceftiofur treated piglets born to ceftiofur + tulathromycin treated sows; C_sow_N_piglet_, non-treated piglets born to ceftiofur treated sows. Each bar represents the microbiota composition in each animal grouped by the study group they belong, where each colour represents one order. Orders under 1% mean relative abundance are summed and represented as “low abundant”. Red color scheme was used for the orders *Burkholderiales* and *Rhizobiales*.

**Additional file 3 Relative abundance of the genera from nasal microbiota of the piglets included in this study including only the ASVs present in the farm core-microbiota (see methods).** Non-treated piglets born to ceftiofur + tulathromycin treated sows (CT_sow_N_piglet_); ceftiofur treated piglets born to ceftiofur + tulathromycin treated sows (CT_sow_C_piglet_); non-treated piglets born to ceftiofur treated sows (C_sow_N_piglet_). Genera with global relative abundance below 0.5% are summed as low abundant.

**Additional file 4 Relative abundance (%) of genera from the piglets’ nasal microbiota that are present in the farm core-microbiota.** Relative abundance of the dominant genera (> 1% global mean) after farm core-microbiota filtering (see methods), shown per sample in the three study groups. CT_sow_N_piglet_**,** non-treated piglets born to ceftiofur + tulathromycin treated sows; CT_sow_C_piglet_, ceftiofur treated piglets born to ceftiofur + tulathromycin treated sows; C_sow_N_piglet_, non-treated piglets born to ceftiofur treated sows. Each bar represents the microbiota composition in each animal grouped by the study group they belong, where each colour represents one genus. Genera under 1% mean relative abundance are summed as low abundant.

**Additional file 5 Beta diversity on Bray–Curtis dissimilarity index for the groups under study.** PCoA was done between CT_**sow**_N_piglet_ (in red) and C_sow_N_piglet_ (in blue) groups in A) and between CT_sow_N_piglet_ (in red) and CT_sow_C_piglet_ (in green) groups in B). CT_sow_N_piglet_**,** non-treated piglets born to ceftiofur + tulathromycin treated sows; CT_sow_C_piglet_, ceftiofur treated piglets born to ceftiofur + tulathromycin treated sows; C_sow_N_piglet_, non-treated piglets born to ceftiofur treated sows. Each dot represents a sample from a piglet. Ellipses of confidence are not shown because of group convergence.

**Additional file 6 Differentially abundant ASVs between CT**_**sow**_**N**_**piglet**_ **and C**_**sow**_**N**_**piglet**_** computed by two different approaches.** ASV taxonomical classification is detailed to the lowest known taxonomical level. The relative abundance in all study groups and the significance in each test is shown per ASV (N.F = not found).

**Additional file 7 Differentially abundant taxa between CT**_**sow**_**N**_**piglet**_** and C**_**sow**_**N**_**piglet**_** groups computed two different approaches.** The relative abundance in all study groups and the significance in each test is shown per ASV (N.F = not found).

## Data Availability

The raw 16S sequences used in this study are available at SRA (NCBI) database under BioProject ID PRJNA990546.
