# Supplementary material for: Intensive antibiotic treatment of sows with parenteral crystalline ceftiofur and tulathromycin alters the composition of the nasal microbiota of their offspring
Source: Vet Res. 2023 Nov 24;54:112. doi: 10.1186/s13567-023-01237-y (PMC10675909; doi:10.1186/s13567-023-01237-y)

Additional File 2. Relative abundance (%) of the top 10 most abundant orders in the piglet's nasal microbiota

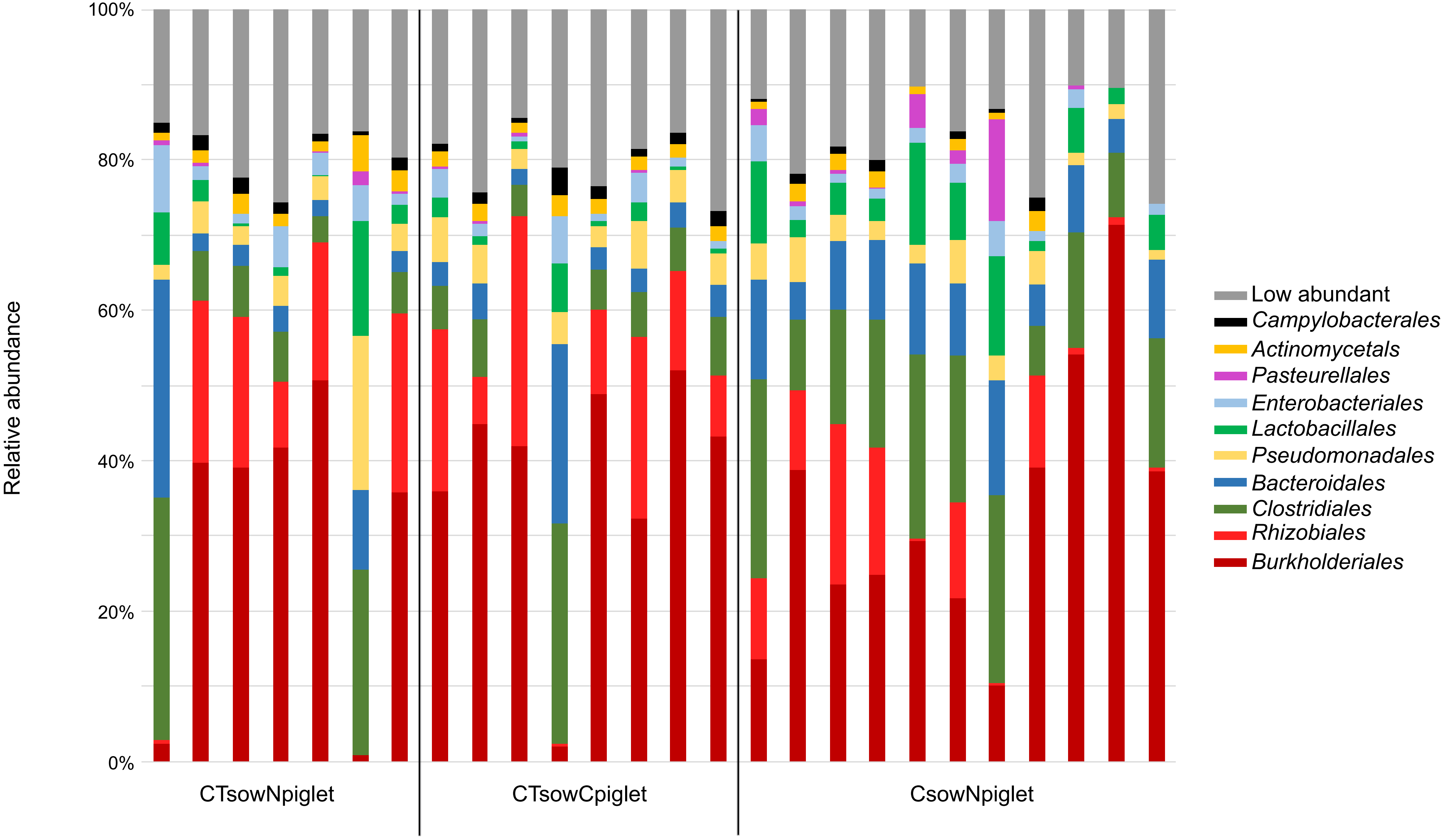

Supplement: Supplementary file 2 — Additional file 2 Relative abundance (%) of the top-10 most abundant orders in the piglet’s nasal microbiota. Microbiota composition is shown for each group included in the present study at order level. CTsowNpiglet, non-treated piglets born to ceftiofur + tulathromycin treated sows; CTsowCpiglet, ceftiofur treated piglets born to ceftiofur + tulathromycin treated sows; CsowNpiglet, non-treated piglets born to ceftiofur treated sows. Each bar represents the microbiota composition in each animal grouped by the study group they belong, where each colour represents one order. Orders under 1% mean relative abundance are summed and represented as “low abundant”. Red color scheme was used for the orders Burkholderiales and Rhizobiales. [file 13567_2023_1237_MOESM2_ESM.pdf]
