# Supplementary material for: Intensive antibiotic treatment of sows with parenteral crystalline ceftiofur and tulathromycin alters the composition of the nasal microbiota of their offspring
Source: Vet Res. 2023 Nov 24;54:112. doi: 10.1186/s13567-023-01237-y (PMC10675909; doi:10.1186/s13567-023-01237-y)

Additional File 4. Relative abundance (%) of genera from the piglets' nasal microbiota that are present in the farm core-microbiota.

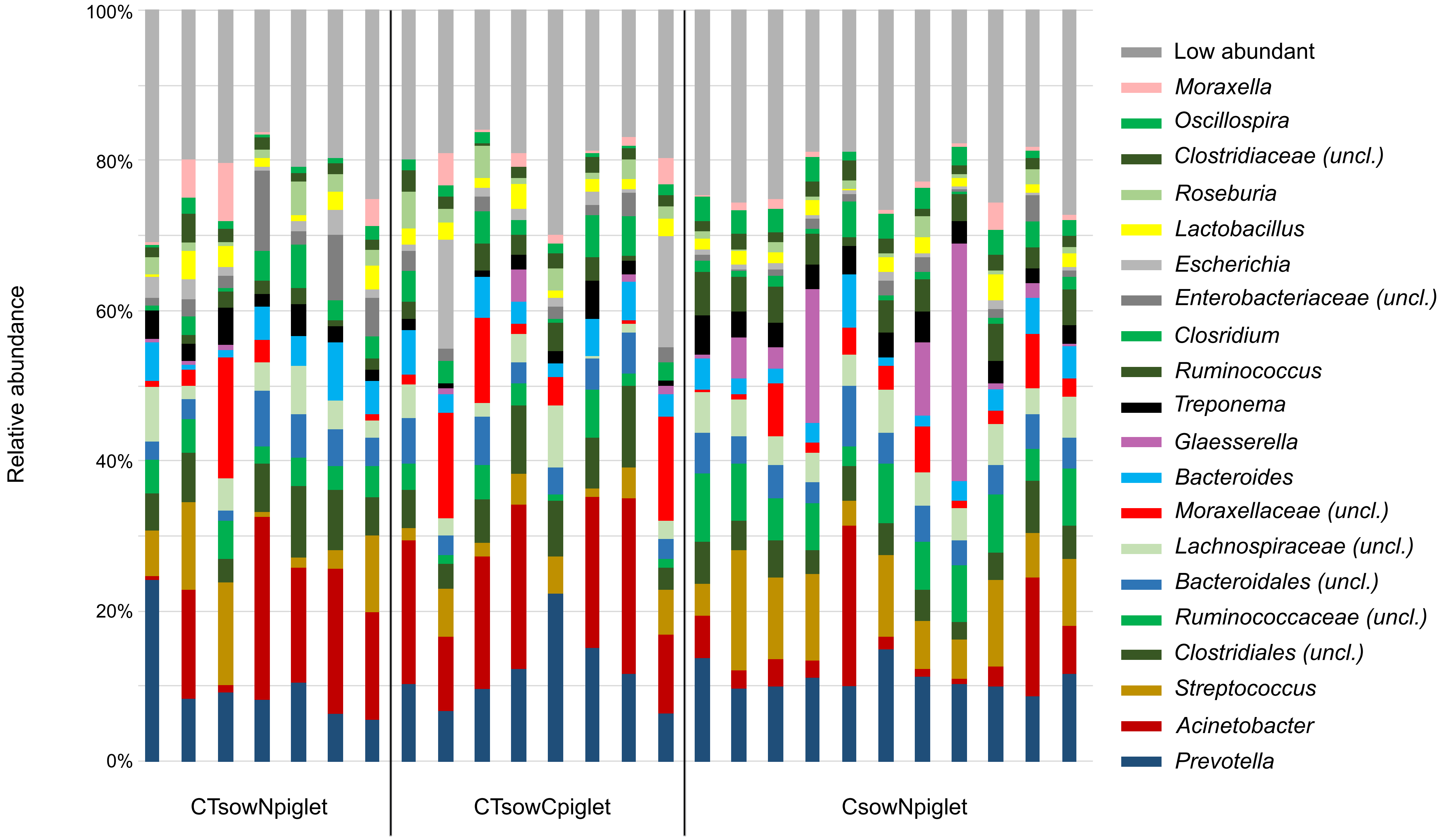

Supplement: Supplementary file 4 — Additional file 4 Relative abundance (%) of genera from the piglets’ nasal microbiota that are present in the farm core-microbiota. Relative abundance of the dominant genera (> 1% global mean) after farm core-microbiota filtering (see methods), shown per sample in the three study groups. CTsowNpiglet, non-treated piglets born to ceftiofur + tulathromycin treated sows; CTsowCpiglet, ceftiofur treated piglets born to ceftiofur + tulathromycin treated sows; CsowNpiglet, non-treated piglets born to ceftiofur treated sows. Each bar represents the microbiota composition in each animal grouped by the study group they belong, where each colour represents one genus. Genera under 1% mean relative abundance are summed as low abundant. [file 13567_2023_1237_MOESM4_ESM.pdf]
