# Supplementary material for: Intensive antibiotic treatment of sows with parenteral crystalline ceftiofur and tulathromycin alters the composition of the nasal microbiota of their offspring
Source: Vet Res. 2023 Nov 24;54:112. doi: 10.1186/s13567-023-01237-y (PMC10675909; doi:10.1186/s13567-023-01237-y)

Additional file 5. Beta diversity on Bray-Curtis dissimilarity index for the groups under study

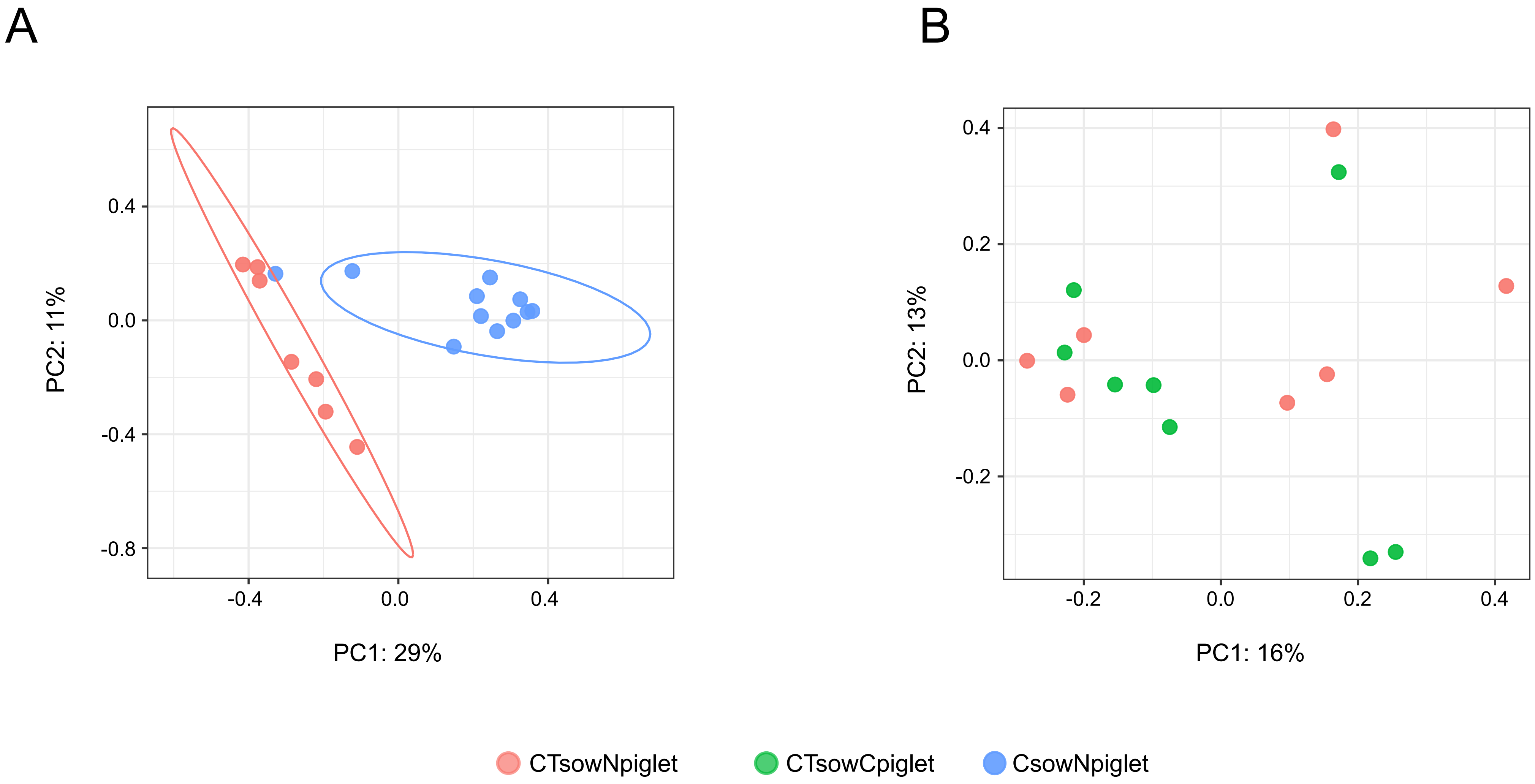

Supplement: Supplementary file 5 — Additional file 5 Beta diversity on Bray–Curtis dissimilarity index for the groups under study. PCoA was done between CTsowNpiglet (in red) and CsowNpiglet (in blue) groups in A) and between CTsowNpiglet (in red) and CTsowCpiglet (in green) groups in B). CTsowNpiglet, non-treated piglets born to ceftiofur + tulathromycin treated sows; CTsowCpiglet, ceftiofur treated piglets born to ceftiofur + tulathromycin treated sows; CsowNpiglet, non-treated piglets born to ceftiofur treated sows. Each dot represents a sample from a piglet. Ellipses of confidence are not shown because of group convergence. [file 13567_2023_1237_MOESM5_ESM.pdf]
